# Connexin-Based Therapeutics and Tissue Engineering Approaches to the Amelioration of Chronic Pancreatitis and Type I Diabetes: Construction and Characterization of a Novel Prevascularized Bioartificial Pancreas

**DOI:** 10.1155/2016/7262680

**Published:** 2015-12-14

**Authors:** J. Matthew Rhett, Hongjun Wang, Heather Bainbridge, Lili Song, Michael J. Yost

**Affiliations:** Department of Surgery, Medical University of South Carolina, Charleston, SC 29425, USA

## Abstract

Total pancreatectomy and islet autotransplantation is a cutting-edge technique to treat chronic pancreatitis and postoperative diabetes. A major obstacle has been low islet cell survival due largely to the innate inflammatory response. Connexin43 (Cx43) channels play a key role in early inflammation and have proven to be viable therapeutic targets. Even if cell death due to early inflammation is avoided, insufficient vascularization is a primary obstacle to maintaining the viability of implanted cells. We have invented technologies targeting the inflammatory response and poor vascularization: a Cx43 mimetic peptide that inhibits inflammation and a novel prevascularized tissue engineered construct. We combined these technologies with isolated islets to create a prevascularized bioartificial pancreas that is resistant to the innate inflammatory response. Immunoconfocal microscopy showed that constructs containing islets express insulin and possess a vascular network similar to constructs without islets. Glucose stimulated islet-containing constructs displayed reduced insulin secretion compared to islets alone. However, labeling for insulin post-glucose stimulation revealed that the constructs expressed abundant levels of insulin. This discrepancy was found to be due to the expression of insulin degrading enzyme. These results suggest that the prevascularized bioartificial pancreas is potentially a tool for improving long-term islet cell survival *in vivo*.

## 1. Introduction

Chronic pancreatitis is a serious inflammatory condition resulting in fibrotic replacement and loss of function of the pancreas. In addition to causing severe pain and discomfort for the patient, left untreated, chronic pancreatitis will result in the inability to produce insulin and subsequent diabetes [1]. Total pancreatectomy and islet autotransplantation (TP-IAT) is a state-of-the-art procedure to treat the intractable pain in patients with chronic pancreatitis and to ameliorate postoperative diabetes [2]. It involves removal of the pancreas, enzymatic digestion of the extracellular matrix, purification of the *β*-cell-containing islets, and reintroduction of the islets to the patient by infusion into the hepatic portal vein, after which the islets take up residence in the liver [2]. However, more than half of islet cells die immediately after intraportal infusion [3, 4]. Although the quality of life is significantly improved in many patients, only about 1/3 of them become insulin independent, 1/3 require minimal insulin replacement (<10 u/day), and the rest develop pancreatogenic diabetes after surgery due to low islet cell survival [2]. Furthermore, even in patients that become insulin independent, many patients become dependent within a period of years [5, 6].

Type 1 diabetes is an autoimmune disease defined by pancreatic *β*-cell death, resulting in insulin deficiency and hyperglycemia [7]. Improved islet cell survival would also beneficially impact allogenic islet transplantation therapy for type 1 diabetes. Unfortunately, pancreatic islet allotransplantation is currently labeled an experimental procedure in the US, and therefore it is generally not covered by health insurance or Medicare [8]. Improving islet cell survival would help to transition the procedure from an experimental to a therapeutic status by increasing the successfulness of the technique.

This issue underlies a common basic challenge facing tissue engineers, namely, the survival of implanted cells and living tissue engineered constructs (TECs)* in vivo*. Implanted devices, cells, and exogenous tissues are often rejected by the patient's body [9]. This can result in serious medical consequences such as physical disfigurement and discomfort, device malfunction, the need for implant removal and tissue reconstruction, and, in the case of TP-IAT, “brittle” diabetes [10–13]. A major factor contributing to poor implanted cell survival is the common host response to implanted devices, TECs, or cells which initially involves coagulation and inflammation, including the infiltration of neutrophils within minutes of the initial injury [14]. Neutrophils are well documented to cause collateral tissue damage during the wound healing response [15–18] and to correlate with increased capsule size and contractility in response to a silicone implant [19]. Importantly, neutrophil infiltration of intraportally introduced islets during the immediate blood mediated inflammatory reaction (IBMIR) has long been documented [20–22]. Additionally, islets can embolize and cause liver necrosis, generating further inflammation [4].

Extracellular ATP (purinergic signaling) is increasingly being recognized as a key proximate signal for inflammation following tissue injury [23, 24] and a major conduit for these signals is connexin proteins [25]. The gap junction (GJ) protein connexin43 (Cx43) is the most ubiquitously expressed isoform of connexin [26]. Cx43 monomers oligomerize to form half-channels (called hemichannels) that are delivered to the plasma membrane [27, 28]. Cx43 hemichannels have been demonstrated to mediate ATP release in a number of scenarios and a number of cell types including vascular endothelial cells [29–35], making hemichannels a prime suspect in the origin of injury-related extracellular ATP signals. Importantly, it has also been shown that purinergic signaling directly activates the clotting response during IBMIR [36].

We and others have shown that manipulation of Cx43 is an effective means to reduce inflammation, accelerate healing, and reduce scarring and capsule formation [15, 17–19]. These studies have culminated in recent efficacious clinical trials for the Cx43 mimetic peptide ACT1 in healing chronic venous leg ulcers and diabetic leg ulcers [37, 38]. We have developed another Cx43 peptidomimetic, named JM2, based on the JuxtaMembrane microtubule binding motif on the Cx43 carboxy-terminus. We have recently demonstrated that JM2 blocks ATP release and strongly inhibits the inflammatory response to a silicone implant in an ATP-dependent manner [39]. Importantly, the Cx43 microtubule binding motif is not conserved between connexin isoforms, making JM2 a specific inhibitor of the Cx43 isoform [40]. Pancreatic *β*-cells solely express Cx36 and are therefore hypothesized to be unaffected by JM2, as opposed to pan-connexin channel blockers that could potentially affect insulin production [41].

Once the initial events of the foreign body response have been negotiated, long-term survival and engraftment of TECs requires host support of the implanted tissue. As stated by Fisher and colleagues in a recent* Tissue Engineering* review article [42], “Complete vascularization of engineered tissues is currently a major hurdle in the field of tissue engineering, inhibiting successful postimplantation viability.” Recently, we have applied a fundamentally different methodology to developing prevascularized tissues. Specifically, 3D endothelial cell-fibroblast constructs were generated in a scaffold-free environment by seeding cells in a nonadherent agarose mold [43, 44]. Importantly, these constructs displayed biophysical properties and histological characteristics of vascular networks, and we are here adopting the nomenclature of self-organizing prevascularized endothelial-fibroblast constructs (SPECs). Crucially, we have recently found that SPECs rapidly anastomose with host vasculature within 3 days and promote tissue repair [45]. In this study, we have combined the two separate, but proven, SPEC and JM2 technologies in a novel configuration to create a prevascularized bioartificial pancreas in order to address a critical need in the treatment of chronic pancreatitis and type 1 diabetes.

## 2. Materials and Methods

### 2.1. Cell Culture and Treatments

Cells were cultured as previously described [44]. Adult normal human dermal fibroblasts (NHDFs) were purchased from Lonza (Walkersville, MD), and human adipose microvascular endothelial cells (HAMECs) were purchased from ScienCell (Carlsbad, CA). The endothelial cells were grown in Medium 200 (M200; Gibco) supplemented with low serum growth supplement (LSGS; Gibco) and 1% penicillin streptomycin solution (PS; Cellgro). Fibroblasts were cultured in high glucose Dulbecco's Modified Eagle's Medium (DMEM; Gibco), supplemented with 10% fetal bovine serum (FBS; Corning), and 1% PS.

JM2 peptide was generated by Petron Inc. (Daejeon, Korea) and was composed, from N-terminus to C-terminus, of a biotin tag, an 8-mer poly-D-arginine internalization vector [46], and the Cx43 C-terminal amino acids 231–245 (VFFKGVKDRVKGRSD) that encompass the microtubule binding sequence (amino acids 234–243 [40]), biotin-rrrrrrrr-VFFKGVKDRVKGRSD. JM2 was solubilized in sterile distilled H_2_O and used at a final concentration of 50 *μ*M. Flufenamic acid (FFA) was purchased from Abcam (Cambridge, MA), diluted in ethanol, and used at a concentration of 50 *μ*M.

### 2.2. Islet Isolation

Islets were isolated from C57BL/6 mice as previously described [47, 48]. Briefly, mice were anesthetized, and the pancreas was perfused with collagenase (type V, 0.6 mg/mL, Sigma Aldrich, St. Louis, MO) through the pancreatic ducts. The dissected enzyme-containing pancreas was then incubated at 37°C with constant shaking to release the islets. Density gradient separation was used to isolate the islets, which were cultured* in vitro* in Dulbecco's Modified Eagles Medium (DMEM; Gibco, Grand Island, NY) plus 10% of fetal bovine serum (Corning, Tewksbury, MA) at 37°C, 5% CO_2_ on low attachment cell culture plates (Corning).

### 2.3. SPEC Fabrication

SPECs were created as previously described [43–45]. Specifically, 7.2 × 10^5^ NHDFs and 1.8 × 10^5^ HAMECs were combined and seeded into nonadherent agarose troughs 9 mm long × 1 mm wide × 1 mm deep. In the case of islet-containing SPECs, 50 islets were combined with the fibroblasts and endothelial cells prior to seeding. The suspensions were cultured for 3 days in a 1 : 2 mixture of M200 (Gibco) media plus LSGS (Gibco) : DMEM (Gibco) plus 10% fetal bovine serum (Corning).

### 2.4. Microscopic Analysis

After 3 days in culture SPECs were either fixed in 4% paraformaldehyde for 1 hr, or cyroembedded in Tissue-Tek O.C.T. Compound (VWR, Radnor, PA). Cryoembedded SPECs were sectioned at 10 *μ*M and fixed in 2% paraformaldehyde. Fixed sections and whole-mount SPECs were labeled with antibodies against insulin (Sigma Aldrich, Cat. number I2018) and CD31 (Abcam, Cat. number ab28364) and labeled for actin using phalloidin (Life Technologies, Grand Island, NY, Cat. number A22284), and the nucleus was labeled with Hoechst stain (Life Technologies, Cat. number H3570). Whole-mount SPECs were imaged on a TCS SP5 laser scanning confocal microscope (LSCM) equipped with 20x/0.75 numerical aperture (NA) and 63x/1.2 NA water objectives (Leica, Buffalo Grove, IL). Sectioned SPECs were also imaged on a TCS SP5 LSCM equipped with a 20x/0.70 NA dry objective and a 63x/1.4 NA oil objective. All figures are maximum projections of z-stacks.

### 2.5. Glucose-Stimulated Insulin Secretion (GSIS) Assay

GSIS assay was performed as previously described [47]. Islets alone, SPECs, and SPECs containing islets were first equilibrated in Krebs buffer (25 mM HEPES, 115 mM NaCl, 24 mM NaHCO_3_, 5 mM KCl, 1 mM MgCl_2_, 0.1% BSA, and 2.5 mM CaCl_2_) with low glucose (2.8 mM) and connexin inhibitors (JM2 or FFA, both used at 50 *μ*M) or vehicle for 2 hrs. The islets/constructs were then treated with Krebs buffer with low glucose (2.8 mM) for 1 hr. The buffer was then collected and replaced with Krebs buffer with high glucose (28 mM) for a second hour, and the buffer was again collected. SPECs with and without islets were then fixed in 4% paraformaldehyde, labeled, and imaged as above. The concentration of insulin released into the buffer was measured using a STELLUX Chemi Rodent Insulin ELISA kit (ALPCO, Salem, NH).

### 2.6. Western Blot

SPECs were homogenized in 250 *μ*L of tricine sample loading buffer (BioRad, Hercules, CA) by shearing with progressively smaller gauge needles beginning with 20 g and ending with 26 g. Confluent cultures of NHDFs and HAMECs were lysed in an equivalent volume of loading buffer using a cell scraper. Samples were resolved by 4–15% SDS-PAGE (BioRad), followed by immunoblotting for insulin degrading enzyme (Abcam, Cat. number 25733) and *α*-tubulin (Sigma Aldrich, Cat. number T8203).

### 2.7. Statistics

Statistics were performed in Prism 5, Version 5.0f (GraphPad Software, Inc., La Jolla, CA). Two-way ANOVA with Bonferroni post hoc test was used to compare the interaction effect between glucose concentration and treatment on insulin secretion, and direct comparisons of the concentration of secreted insulin within treatment groups from low to high glucose were made by paired *t*-tests. One-way ANOVA with Bonferroni post hoc test was used to compare the fold change in insulin secretion from low to high glucose.

## 3. Results

### 3.1. Insulin Expression and Vascular Network Formation Are Maintained in Islet-Containing SPECs

**Figure 1:**
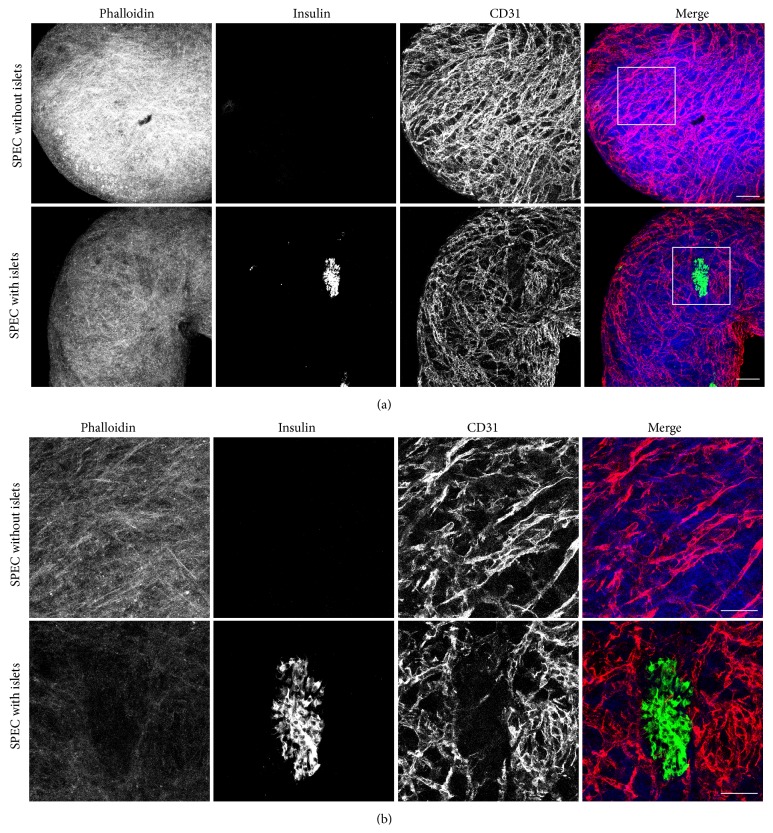
Incorporation of islets into SPECs does not affect vascular network formation. SPECs with and without islets were generated, fixed, and whole-mount-labeled for actin (phalloidin, blue), islets (insulin, green), and endothelial cells (CD31, red). The images are maximum projections of z-stacks acquired by confocal microscopy under 20x magnification (a) and 63x magnification [(b), boxed regions in (a)]. Note the dense, web-like pattern of endothelial cells in both constructs, indicating a robust vascular network. Importantly, the vascular network surrounds the embedded islet. Scale bars represent 100 *μ*m and 50 *μ*m in (a) and (b), respectively.

**Figure 2:**
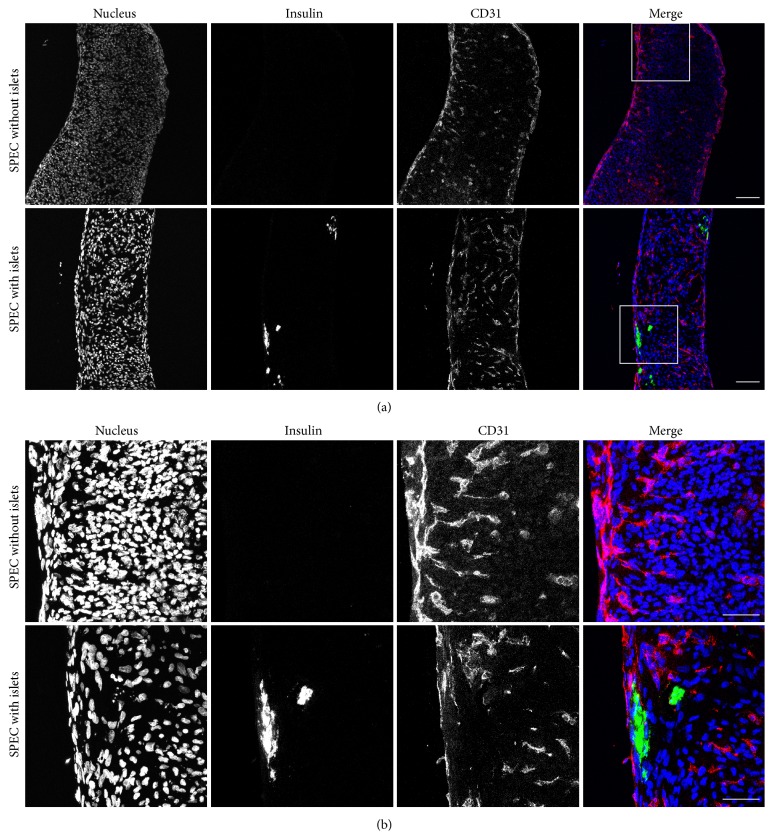
Islets seeded into SPECs embed throughout the body of the construct. Cryoembedded SPECs with and without islets were sectioned, fixed, and labeled for nuclei (blue), islets (insulin, green), and endothelial cells (CD31, red). The images are maximum projections of z-stacks acquired by confocal microscopy under 20x magnification (a), and 63x magnification [(b), boxed region in (a)]. Islets can be seen both in the periphery and the interior of the islet-containing SPECs. It is interesting to note that the vascular network extends to the interior of the construct and that this is also not disrupted by the presence of islets. Scale bars represent 100 *μ*m and 50 *μ*m in (a) and (b), respectively.

Our first goal was to determine the effects of seeding islets together with endothelial cells (HAMECs) and fibroblasts (NHDFs) as previously described to fabricate SPECs [43]. Specifically, we wanted to determine if (a) the islets would be included or excluded from the SPECs and (b) if inclusion of islets would affect vascular network formation, as it has been shown that altering the ratio of HAMECs : NHDFs affects the vascular network [44]. To that end, we constructed SPECs with the previously determined optimal ratio of 1 : 4 HAMECs : NHDFs, and in half of the SPECs we added 50 islets per construct. These constructs were then fixed and labeled for insulin as a marker for islets [47] and CD31 as a marker for endothelial cells [49]. In whole-mount SPECs, insulin-expressing islets were observed to be embedded in the exterior of the SPECs (Figure 1). Furthermore, we also found islets in the interior of SPECs in fresh-frozen tissue sections (Figure 2). Importantly, a vascular network was discernable on the surface of SPECs both with and without islets (Figure 1). In tissue sections, this network could be seen extending to the interior of SPECs irrespective of the presence of islets (Figure 2). Taken together, these results demonstrate that SPECs incorporate islets without affecting vascular network formation, and the *β*-cells in the incorporated islets continue to express insulin.

### 3.2. Prevascularized Bioartificial Pancreases Display Reduced Insulin Secretion

**Figure 3:**
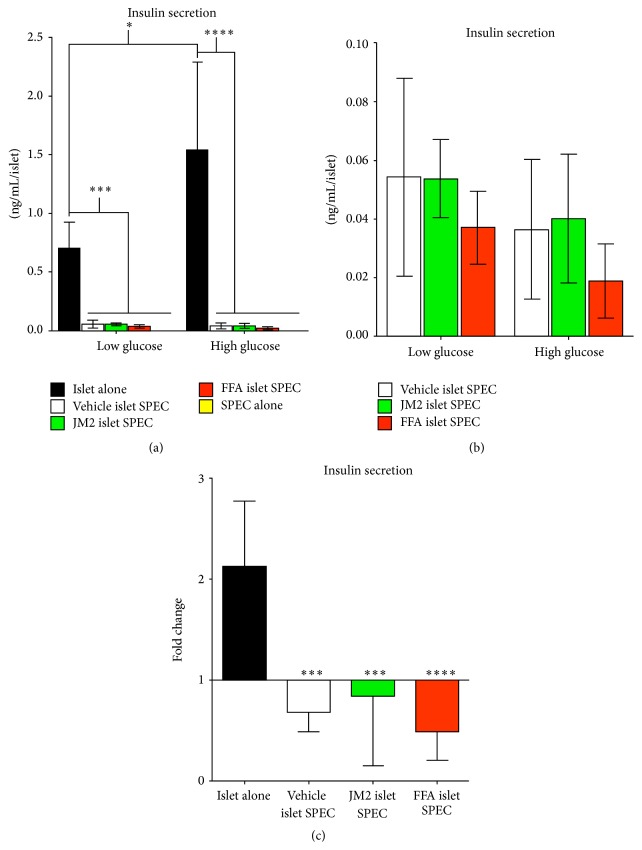
Islet-containing SPECs secrete low levels of insulin and fail to respond to high glucose stimulation. Islets alone, SPECs without islets, and islet-containing SPECs pretreated with vehicle, 50 *μ*M JM2, or 50 *μ*M FFA were subjected to GSIS assay by first stimulating with low glucose (2.8 mM) for one hour and then high glucose (28 mM) for one hour. Buffer was collected at the end of both stimulation periods and analyzed for insulin by ELISA. (a) The concentration of insulin secreted per islet for all treatment groups. Two-way ANOVA revealed that there was significant pairing (*P* < 0.01) and that glucose concentration and treatment were both significant sources of variation (*P* < 0.0001 and *P* < 0.001, resp.). Bonferroni post hoc analysis determined that within the low glucose group free islets alone secreted significantly more insulin than any of the islet-containing SPEC treatments (*P* < 0.001, indicated on graph by *∗∗∗*) and that, similarly, high glucose free islets secreted significantly more insulin than any of the high glucose islet-containing SPEC treatments (*P* < 0.0001, indicated on graph by *∗∗∗∗*). Paired *t*-tests were used to compare high and low glucose conditions within treatment groups, and only free islets displayed a significant increase in insulin secretion upon high glucose stimulation (*P* < 0.01, indicated on graph by *∗*). (b) The graph in (a) was limited to comparisons between islet-containing SPECs to better resolve the graph bars. No significant differences were observed. (c) The relative change in insulin secretion from low to high glucose stimulation. One-way ANOVA with Bonferroni post hoc analysis showed that the islets alone had a significantly increased relative change in insulin secretion compared to any of the SPEC treatment groups (*P* < 0.001). These data show that islet-containing SPECs secrete very low levels of insulin and that they are refractory to high glucose stimulation.

We next tested whether islet-containing SPECs, or prevascularized bioartificial pancreases, secreted insulin and responded to high glucose challenge by GSIS assay. Islets alone, SPECs without islets, and islet-containing SPECs pretreated for 2 hrs with ether vehicle, 50 *μ*M JM2, or 50 *μ*M FFA were first subjected to low glucose (2.8 mM) for 1 hr and then high glucose (28 mM) for a second hour. Following high glucose challenge, the constructs were fixed and the low and high glucose buffers were analyzed for insulin concentration by ELISA. Islets alone displayed normal function, producing nanogram levels of insulin per islet (Figure 3(a)) and responding to high glucose by significantly doubling insulin secretion (Figures 3(a) and 3(c)). In contrast, the prevascularized bioartificial pancreases secreted very low levels of insulin that were significantly less than islets alone (Figure 3(a)). Surprisingly, islet-containing SPECs were not only unresponsive to high glucose but even displayed a trend toward reduced insulin secretion (Figure 3(b)), although this effect was not statistically significant. However, when compared to islets alone, the response to high glucose by islet-containing SPECs was significantly reduced (Figure 3(c)).

In order to gain further insight, we limited the statistical comparisons to prevascularized bioartificial pancreases. Two-way ANOVA revealed that there was a significant effect by treatment group. Consistent with this, we noted that FFA appeared to further suppress insulin secretion relative to vehicle and JM2-treated islet-containing SPECs. However, Bonferroni post hoc analysis failed to confirm that this was a statistically significant effect (Figure 3(b)). These data suggest that insulin expression or secretion by islets incorporated in prevascularized bioartificial pancreases was inhibited. Alternatively, the fibroblast and endothelial cells of the SPEC may have sequestered or degraded the secreted insulin.

### 3.3. Insulin Expression Is Maintained in JM2-Treated, Prevascularized Bioartificial Pancreases

**Figure 4:**
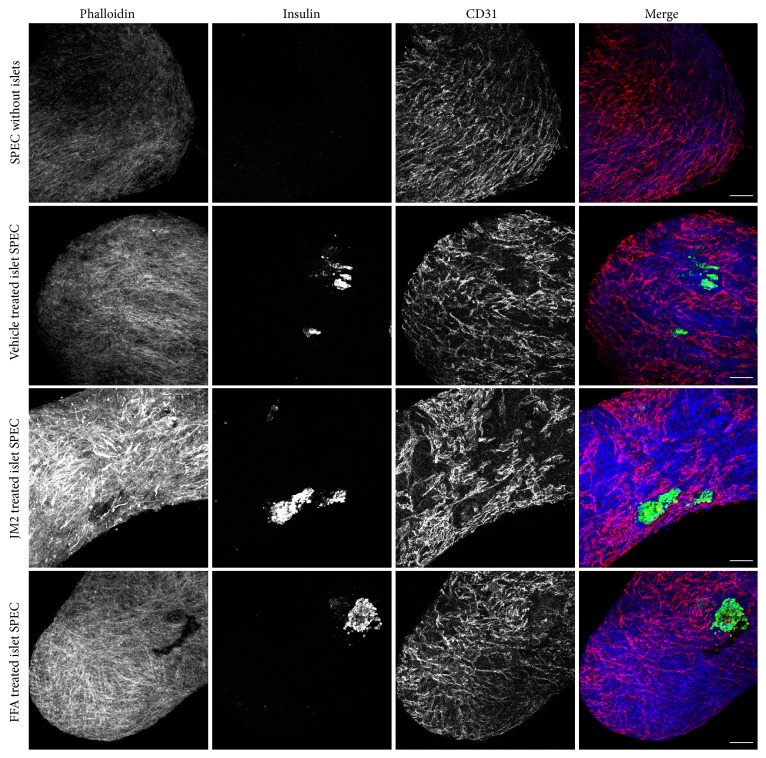
Prevascularized bioartificial pancreases subjected to JM2 treatment retain robust insulin expression and maintain a vascular network. The constructs used in the GSIS assay were fixed, whole-mount-labeled, and imaged by confocal microscopy. The labels used were for actin (phalloidin, blue), islets (insulin, green), and endothelial cells (CD31, red). Note the persistence of a vascular network in the constructs subjected to GSIS assay (Vehicle, JM2, and FFA). Importantly, the level of insulin expressed is comparable in vehicle and JM2-treated constructs, while this appears to be decreased in FFA-treated SPECs. Images are maximum projections of z-stacks of 20x magnification fields. Scale bars represent 100 *μ*m.

We explored the observed lack of secreted insulin by immunolabeling the SPECs containing islets and SPECs without islets subjected to GSIS assay. As above, the constructs were labeled with antibodies against insulin and CD31 to identify islets and endothelial cells, respectively. All constructs displayed an intact vascular network (Figure 4), indicating that neither the GSIS assay nor the connexin inhibitors affected maintenance of the vascular network. Unexpectedly and in contrast to the level of insulin secretion, insulin expression by islets in vehicle and JM2-treated prevascularized bioartificial pancreases was comparable to constructs imaged without being subjected to GSIS assay (Figure 4). To confirm that this level of insulin expression was comparable to free islets, sectioned islet-containing SPECs and free islets were labeled for insulin, and no differences were observed (Supplemental Figure  1) (see Supplementary Material available online at http://dx.doi.org/10.1155/2016/7262680). In contrast, consistent with the trend toward reduced insulin secretion observed for FFA-treated prevascularized bioartificial pancreases, FFA-treated constructs displayed reduced insulin expression post-GSIS assay (Figure 4).

Due to the reduced insulin secretion observed in islet-containing SPECs (Figure 3) and findings that show that while Cx36 is the predominant isoform Cx43 is also expressed in islets [50], we sought to directly test whether or not JM2 and FFA affect insulin secretion. As such, free islets pretreated with JM2 and FFA were subjected to GSIS assay. We found that vehicle and JM2-treated islets responded normally to high glucose, approximately doubling their insulin secretion (Supplemental Figure  2). Consistent with the results of the GSIS assay performed on islet-containing SPECs and the associated post-GSIS assay labeling, FFA failed to increase insulin secretion in response to high glucose (Supplemental Figure  2). Also consistent with the results of the GSIS assay performed on islet-containing SPECs, two-way ANOVA revealed that there was a significant effect by treatment group. Together, these results indicated that neither the presence of islets, GSIS assay, nor connexin inhibitors affected vascular network formation. However, pan-connexin blockers, like FFA, inhibit insulin expression and/or secretion, while the targeted inhibitor JM2 did not. Additionally, incorporation of islets into SPECs inhibited insulin secretion.

### 3.4. SPECs Express Insulin Degrading Enzyme

**Figure 5:**
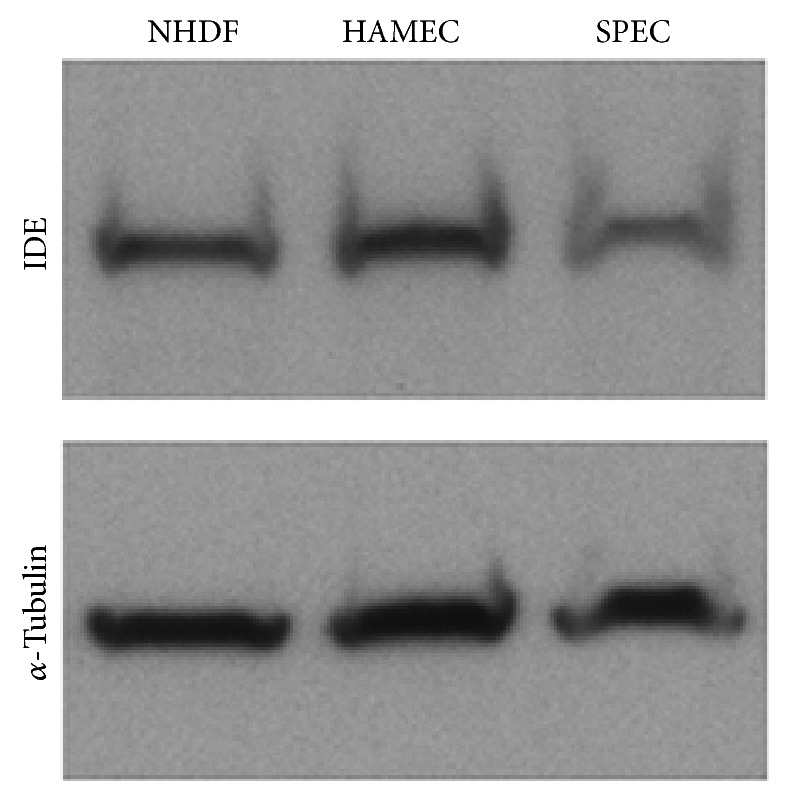
Prevascularized bioartificial pancreases express insulin degrading enzyme. SPECs, fibroblasts (NHDFs), and endothelial cells (HAMECs) were homogenized in sample loading buffer, resolved by SDS-PAGE, and immunoblotted for insulin degrading enzyme (IDE) and *α*-tubulin. Robust expression of insulin degrading enzyme was observed both in the cultured cells and in the assembled construct.

Notably, we did not detect insulin sequestered or “trapped” within the body of islet-containing SPECs. In combination with the data indicating that insulin expression was not affected by vehicle or JM2 treatment in islet-containing SPECs (Figure 4 and Supplemental Figure  1) and insulin secretion was not affected by vehicle or JM2 treatment in free islets (Supplemental Figure  2), this suggested that insulin may be degraded in insulin-containing SPECs. Therefore, we tested for the presence of insulin degrading enzyme in SPECs. To this end, SPECs were homogenized in sample loading buffer and 2D cultures of HAMECs and NHDFs were lysed in sample loading buffer. Western analysis of the samples showed that insulin degrading enzyme was robustly expressed in both cultures of primary cells as well as the SPEC constructs themselves (Figure 5). These results indicate that insulin secreted by islets embedded in SPECs may be degraded by insulin degrading enzyme expressed by the other component cells of the construct.

## 4. Discussion

To our knowledge, this is the first demonstration of a prevascularized artificial organ constructed with both parenchymal cells and stromal cells using scaffold-free technology (Figures 1 and 2). The novelty of this finding stems directly from the delicate and highly orchestrated nature of vascular network formation. A number of studies have shown that the formation of a vascular network is dependent on the combination of the presence of a balanced cocktail of growth factors with an appropriate environmental context of specific extracellular matrix molecules and stromal cells [44, 51–54]. Therefore it would have been unsurprising if the addition of parenchymal cells negatively impacted the ability of the construct to form vascular networks.

This is a particularly important finding in the context of transplanted islet cells.* In vivo*, pancreatic islets are normally highly vascularized structures, which is crucial for proper function [55]. Using current protocols, transplanted islets may not become vascularized for a period of days to weeks [56]. Importantly, the robust SPEC vascular network displayed a resemblance to the vasculature of pancreatic islets* in vivo* (e.g., compare Figure 1 with the live,* in vivo* imaging of Powers and coworkers [57]). Given the ability of SPECs to rapidly anastomose with host vasculature [45], this work represents a major step forward in developing the ability to more effectively transplant isolated islets.

Equally important was the finding that connexin inhibitors did not affect the integrity of the already formed microvasculature (Figure 4). This was somewhat unexpected in that connexins are important in cell migration and vascular network formation [58–60]. However, the SPECs were only exposed to JM2 and FFA for a relatively short period of time (2 hrs), and it is unknown if longer exposures would have a different effect on the vascular network. We and others have previously shown that targeted inhibition of Cx43 channels improves wound healing and the response to implanted materials [15, 18, 19, 38, 39, 61]. These effects appear to be mediated through dampening of purinergic signals that attract neutrophils to injury sites [25, 39]. The significance of this observation is that only a limited window of exposure to connexin-based therapeutics is necessary to reduce tissue damage caused by the innate inflammatory response. Therefore, our results suggest that the acute treatment of an implant site with targeted Cx43 inhibitors could improve integration of artificial tissues (such as the islet-containing SPECs described herein) without disrupting a preformed vascular network.

In addition to a lack of effect on vascular network formation, the targeted Cx43 inhibitor JM2 did not affect insulin expression. In contrast, reduced insulin levels were observed in FFA-treated SPECs (Figures 3 and 4 and Supplemental Figure  2). This highlights a benefit of targeted connexin inhibitors such as JM2 and ACT-1. Namely, while pan-connexin inhibitors like FFA and mefloquine do inhibit hemichannel function and dampen the inflammatory response [39, 62], they can also have unintended side effects such as effects on insulin expression [41, 63, 64]. Connexin-mimetic peptides such as JM2 and ACT-1 are short-lived and have limited side effects even when administered systemically [65, 66].

Unexpectedly, we found that islets incorporated in SPECs displayed reduced insulin secretion (Figure 3). Labeling of the constructs post-GSIS assay suggest that the constructs themselves were not sequestering insulin secreted by the islets because insulin labeling was restricted to the islets (Figure 4). This left the possibilities that the other cells in the construct degraded the insulin, or that insulin secretion was inhibited by interactions between the *β*-cells of the islets and the cells making up the rest of the SPEC. Insulin degrading enzyme has been shown to be expressed by a number of cell types including endothelial cells [67]. Importantly, it has been localized to the cell surface of cerebrovascular endothelial cells [68] and is secreted by a number of other cell types [69]. Consistently, our results show that both the endothelial cells and fibroblasts used in constructing the SPECs robustly express insulin degrading enzyme, and this expression is maintained in assembled SPECs (Figure 5). Therefore, it is likely that the apparent level of insulin secreted by our prevascularized bioartificial pancreases was low due to degradation by insulin degrading enzyme expressed by the endothelial cells and/or fibroblasts in the construct.

Alternative strategies to improving insulin secretion may therefore need to be implemented before our prevascularized bioartificial pancreas technology can be implemented. One such approach would involve gene targeting in order to knock down the expression of insulin degrading enzyme. For example, the CRISPR/Cas9 system is a new technology that has the power to rapidly and efficiently edit the genome of cultured cells in a targeted manner [70], and this technique could be employed to block the expression of insulin degrading enzyme in our constructs.

Another strategy may be to employ different cell types and/or a higher ratio of islets to construct cells. A similar approach to ours has been previously attempted by Johansson et al. in which isolated islets were “coated” with endothelial cells and mesenchymal stem cells, and the constructs were found to secrete insulin at the same level as control free islets [71]. In Johansson and coworker's study mesenchymal stem cells and dermal endothelial cells were used as opposed to dermal fibroblasts and adipose-derived endothelial cells in our study. It is possible that mesenchymal stem cells and dermal endothelial cells express lower levels of insulin degrading enzyme than the NHDFs and HAMECs used in our study. In addition, the islets used in the Johansson et al. study were merely coated with the construct cells [71], while our islets were embedded in a larger construct. Therefore, even if the mesenchymal stem cells and endothelial cells used by Johansson et al. express levels of insulin degrading enzyme comparable to the cells in our constructs, there would be less insulin degrading enzyme present relative to the level of insulin produced in their constructs. Thus a possible solution to improving insulin secretion by our constructs would be to increase the number of islets incorporated per construct; however, there is likely a critical threshold of islets that can be embedded in each SPEC that will prevent vascular network formation and/or reduce the constructs' anastomotic potential. This is a critical point because while Johansson and coworker's constructs did appear to form “vascular sprouts,” our approach differs significantly in that the islets in our constructs are embedded in a much larger vascularized structure that has been demonstrated to anastomose with host vasculature when implanted [45]. Additionally, immunoisolation is enhanced in our prevascularized bioartificial pancreases by incorporating the anti-inflammatory peptide, JM2, which could be “loaded” into the constructs by pretreating the islet-containing SPECs prior to implantation [39].

Another aspect of the Johansson et al. study that differed from ours was the use of a dynamic perfusion GSIS assay [71]. By perfusing the constructs, the media is continuously sampled, and therefore insulin secreted by the their constructs may have been collected before it could be degraded by the construct cells. In the GSIS assay we employed, free islets or constructs were bathed in static high and low glucose buffers, which were collected at the end of an hour. Thus, our static conditions would cause any insulin secreted by our constructs to be continuously exposed to the insulin degrading enzyme-expressing construct cells during the hour-long incubations, resulting in degradation of the majority of the secreted insulin. If this is the case, then the constructs we developed may not need any modification because* in vivo* the job of perfusion would be carried out by the host vasculature. In other words, rapid anastomosis of the host vasculature with that of our prevascularized bioartificial pancreases would be sufficient to transport the insulin secreted by our constructs to the proper destination tissues before the insulin could be degraded.

The concept of the bioartificial pancreas has existed since the 1980s [72]. The principal behind the bioartificial pancreas is that islets encapsulated in a microsphere of hydrogel material (typically alginate) will be “immunoisolated” (i.e., protected from the immune system) but maintain the ability to take in oxygen and nutrients and secrete insulin [73]. However, clinical implementation of this technology has been hampered by lack of revascularization of these constructs [55]. The prevascularized bioartificial pancreases described herein provide a potential solution to this critical hurdle common to the tissue engineering field in general [42].

## 5. Conclusions

Connexin-based peptides are gaining headway as potential therapeutics with novel applications from the prevention of arrhythmia to cancer treatments [18, 37, 38, 61, 65, 66, 74, 75]. We have here combined it with a novel technology to promote vascularization and perfusion of implanted tissue engineered constructs and isolated islets to create a prevascularized bioartificial pancreas. These constructs may eventually provide a more viable alternative to current islet transplant procedures to meet an urgent need for long-term solutions to type 1 diabetes and postoperative “brittle” diabetes subsequent to pancreatectomy.

## Supplementary Material

Figure 1: Demonstrates that insulin levels are similar in free islets and islets embedded in SPECs.
Figure 2: Shows that while the broad-spectrum connexin inhibitor flufenamic acid inhibits insulin secretion, JM2 does not affect insulin secretion in free islets.
